# Supplementary material for: Physicochemical Characteristics of Soluble Dietary Fiber Obtained from Grapefruit Peel Insoluble Dietary Fiber and Its Effects on Blueberry Jam
Source: Foods. 2022 Nov 21;11(22):3735. doi: 10.3390/foods11223735 (PMC9689297; doi:10.3390/foods11223735)
Supplement: Supplementary file 1 [file foods-11-03735-s001.zip › foods-1987290-supplementary.pdf]

Supplementary Material

Physicochemical Characteristics of Soluble Dietary Fiber Obtained from Grapefruit Peel Insoluble Dietary Fiber and Its effects on Blueberry Jam

Jiayan Xie, Guanyi Peng, Qiang Yu, Jianhua Xie, Yi Chen, Ruihong Dong, Jingyu Si, Chaoran Yang, Xiaobo Hu*

Supplementary Tables S1–S2

Table S1 Formulation of blueberry jams.

Ingredients used to make jam	Amount (g, ingredients of 100 g blueberry pulp)			
	BJ	LMJ	GPSJ-1	GPSJ-2
Sucrose	30	30	30	30
Citric acid	0.1	0.1	0.1	0.1
LM pectin	-	1.0	-	0.5
$CaCl_2$	0.3	0.3	0.3	0.3
GP-IDF-SDF	-	-	1.0	0.5

Note: BJ, basic jam prepared with blueberry pulp, sucrose and citric acid; LMJ, jam prepared with blueberry pulp, sucrose, citric acid and 1% LM pectin; GPSJ-1, jam prepared with 1% GP-IDF-SDF; GPSJ-2, jam prepared with 0.5% GP-IDF-SDF and 0.5% LM pectin.

Table S2 Monosaccharides composition ratio of GP-IDF-SDF.

Monosaccharide	Ratio
Fructose (Fru)	ND
Rhamnose (Rha)	ND
Arabinose (Ara)	3.86
Galactose (Gal)	1
Glucose (Glu)	3.04
Xylose (Xly)	2.19
Mannose (Man)	1.11
Galacturonic acid (Gala)	6.06

Note: ND, not detected. Ratio means the amount of different monosaccharides in different samples comparted to the amount of mannose in GP-IDF-SDF.
